# Why catheter size matters more than viscosity in ethylene–vinyl alcohol copolymer reflux: an analytical study

**DOI:** 10.1186/s42155-026-00693-9

**Published:** 2026-05-20

**Authors:** Arthur A. Cornelis, Ruben Geevarghese, Stephen B. Solomon, Francois H. Cornelis

**Affiliations:** 1 Yale University, 38 Hillhouse Av, New Haven, CT 06511 USA; 2 Department of Radiology, Memorial Sloan Kettering Cancer Center, 1275 York Avenue, New York, NY 10065 USA; 3 Weill Cornell Medical College, 1300 York Avenue, New York, NY 10065 USA

**Keywords:** Liquid embolics, Ethylene–vinyl alcohol copolymer, Endovascular embolization, Interventional radiology, Computer simulation

## Abstract

**Purpose:**

Proximal reflux of liquid embolic agents remains a primary safety concern during endovascular embolization, yet the quantitative relationships governing reflux mechanics are poorly characterized. An analytical model was developed to determine the relative contributions of catheter geometry, agent viscosity, polymerization kinetics, and injection technique to reflux behavior of ethylene–vinyl alcohol copolymer (EVOH).

**Methods:**

An annular Poiseuille flow model was constructed for a 1 mm vessel with coaxial microcatheters (1.3–2.8 French; catheter-to-vessel diameter ratio D* = 0.43–0.92). Carreau rheology was applied to EVOH-18 and EVOH-34; blood and iodinated contrast were modeled as Newtonian. Extensions incorporated vessel compliance, first-order EVOH polymerization kinetics, and intermittent injection parameterized by duty cycle δ.

**Results:**

The annular geometry function φ(D*) spanned three orders of magnitude across the catheter range (0.179 to 0.0006), surpassing the maximum 2.4-fold inter-agent viscosity difference. At injection shear rates, EVOH-18 thinned to near-contrast viscosity (6.4 vs 6.1 mPa·s). EVOH polymerization reduced reflux up to 68% at low D* but was negligible at D* > 0.9. Under intermittent injection (δ = 0.20), effective reflux lengths fell up to tenfold, and the protective advantage of EVOH-34 over contrast increased from 1.35-fold to 4.1-fold.

**Conclusions:**

Catheter-to-vessel geometry dominates reflux by orders of magnitude over viscosity; catheter selection is the primary modifiable safety determinant. EVOH polymerization provides geometrically selective secondary protection maximized by intermittent injection. The duty cycle δ is proposed as a standardizable parameter for reporting injection protocols.

**Supplementary Information:**

The online version contains supplementary material available at 10.1186/s42155-026-00693-9.

## Introduction

Liquid embolic agents based on ethylene–vinyl alcohol copolymer (EVOH) dissolved in dimethyl sulfoxide (DMSO) are widely used for endovascular treatment of arteriovenous malformations, dural arteriovenous fistulas, and tumors [1, 2]. Proximal reflux along the catheter remains a primary safety concern, risking non-target embolization, catheter entrapment, and incomplete nidal penetration [3]. Higher-viscosity formulations such as EVOH-34 are empirically preferred when reflux risk is elevated, yet the quantitative relationship between fluid properties, catheter geometry, and reflux dynamics has not been systematically modeled [4, 5]. We hypothesized that catheter-to-vessel diameter ratio (D*) dominates reflux mechanics over fluid viscosity. This study is coupling annular Poiseuille flow with Carreau rheology to compare reflux behavior across four clinically relevant fluids, extended to incorporate vessel compliance, EVOH polymerization kinetics, and intermittent injection protocols.

## Methods

The full methodology is provided as supplemental material. A rigid cylindrical vessel (diameter 1 mm) was modeled with coaxial microcatheters from 1.3 to 2.8 French (D* = 0.43–0.92). Blood and iodinated contrast (Iohexol 300) were treated as Newtonian fluids (3.5 and 6.1 mPa·s). Although blood is a well-characterized non-Newtonian fluid exhibiting shear-thinning and viscoelasticity [6, 7], at the wall shear rates in this model (> 1,000 s⁻^1^) its apparent viscosity approaches the high-shear asymptotic plateau, making the Newtonian approximation at 3.5 mPa·s both established and conservative: it represents a viscosity lower bound that maximizes predicted reflux. EVOH-18 and EVOH-34 followed the Carreau model (EVOH-18: μ₀ = 40, μ∞ = 6 mPa·s, λ = 2.5 s, n = 0.45; EVOH-34: μ₀ = 80, μ∞ = 8 mPa·s, λ = 3.5 s, n = 0.40) [8–11]. Baseline blood flow was 1.0 mL/min with injection at 0.3 mL/min. The dimensionless annular geometry function φ(D*), which quantifies how easily fluid flows backward through the catheter–vessel gap, governed annular flow conductance. A reflux ratio threshold of Q* ≤ 0.05 defined maximum safe injection pressure; a reference reflux length of 5 mm was used for pressure calculations. Both are theoretical model ceilings, not clinical targets. Three extensions were evaluated: vessel compliance via a linear pressure-diameter relationship; time-dependent EVOH viscosity augmentation from precipitation modeled as a first-order process (K_p = 0.05–0.20 s⁻^1^); and intermittent injection parameterized by duty cycle δ = t_inject/(t_inject + t_pause), the fraction of time spent actively injecting during a push-and-wait cycle [3].

## Results

### Effective viscosity

At injection shear rates (~ 1,200 s⁻^1^), EVOH-18 thinned to 6.4 mPa·s, indistinguishable from contrast (6.1 mPa·s). EVOH-34 thinned to 8.3 mPa·s. Flow was laminar throughout (Re 14–450; Table 1).

**Table 1:** Effective Viscosity (mPa·s) Across Shear Rates

Shear Rate (s⁻^1^)	Blood	Contrast	EVOH-18	EVOH-34
400	3.50	6.10	7.03	8.75
1,200	3.50	6.10	6.42	8.28
2,000	3.50	6.10	6.28	8.18
4,000	3.50	6.10	6.17	8.10
6,000	3.50	6.10	6.13	8.08

Note — Blood and contrast are Newtonian (constant viscosity). EVOH values computed via Carreau model with iterative shear-rate resolution

### Catheter size dominates

The φ(D*) decreased ~ 300-fold from D* = 0.43 to 0.92 (0.1786 to 0.0006), far exceeding inter-fluid viscosity differences (≤ 2.4 ×). Predicted reflux lengths at 50 psi ranged from hundreds of millimeters at low D* to sub-millimeter near wedge. The Craya–Curtet number (Ct), which compares the forward momentum of native blood flow to that of the injected embolic, exceeded 2 at all D* values, confirming that blood momentum actively opposed reflux (Tables 2 and 3).

**Table 2:** Annular Geometry Function and Conductance by Catheter Size

Catheter (F)	D*	φ(D*)	Annular Gap (mm)
1.3	0.43	0.1786	0.285
1.5	0.50	0.1259	0.250
1.7	0.56	0.0892	0.220
2.0	0.66	0.0437	0.170
2.4	0.79	0.0110	0.105
2.8	0.92	0.0006	0.040

Note — D* = catheter-to-vessel diameter ratio; φ(D*) = dimensionless annular geometry function governing flow conductance.*

**Table 3:** Craya–Curtet Number (Q_b = 1.0 mL/min, Q_c = 0.3 mL/min). The Craya–Curtet number (Ct) quantifies the ratio of blood momentum to injection jet momentum in the annular space; values exceeding 1.0 indicate that native blood flow dominates the annular hemodynamics over the injected agent. All Ct values in this model exceeded 2.2 across the full catheter range, confirming that blood momentum consistently opposes proximal reflux — a physiological self-limiting mechanism that partially counteracts injection-driven retrograde flow. Higher Ct values at larger D* (e.g., 7.44–7.55 at D* = 0.92) indicate progressively stronger blood-flow resistance to reflux as the annular gap narrows, explaining in part why near-wedge positioning is so protective even at high injection pressures

D*	Contrast	EVOH-18	EVOH-34
0.43	2.21	2.24	2.24
0.50	2.47	2.50	2.50
0.56	2.75	2.79	2.79
0.66	3.29	3.34	3.34
0.79	4.41	4.47	4.47
0.92	7.44	7.55	7.55

Note — Ct > 1 indicates blood momentum dominance in annular space. Blood values are identical to EVOH-18 and omitted for clarity

### Vessel compliance

Compliance effects were modest at low-to-moderate D* (< 35% increase) but amplified near wedge: at D* = 0.92, medium compliance increased φ 7.3-fold, raising EVOH-34 reflux from 0.60 to 4.4 mm (Tables S1–S2).

### Polymerization

Polymerization most benefited low D*, where long reflux columns allowed sufficient blood contact for viscosity augmentation (EVOH-34 reflux reduced ~ 68% at D* = 0.43). At D* ≥ 0.79, contact times were short and polymerization had minimal impact (Table S3).

### Intermittent injection

**Fig. 1:**
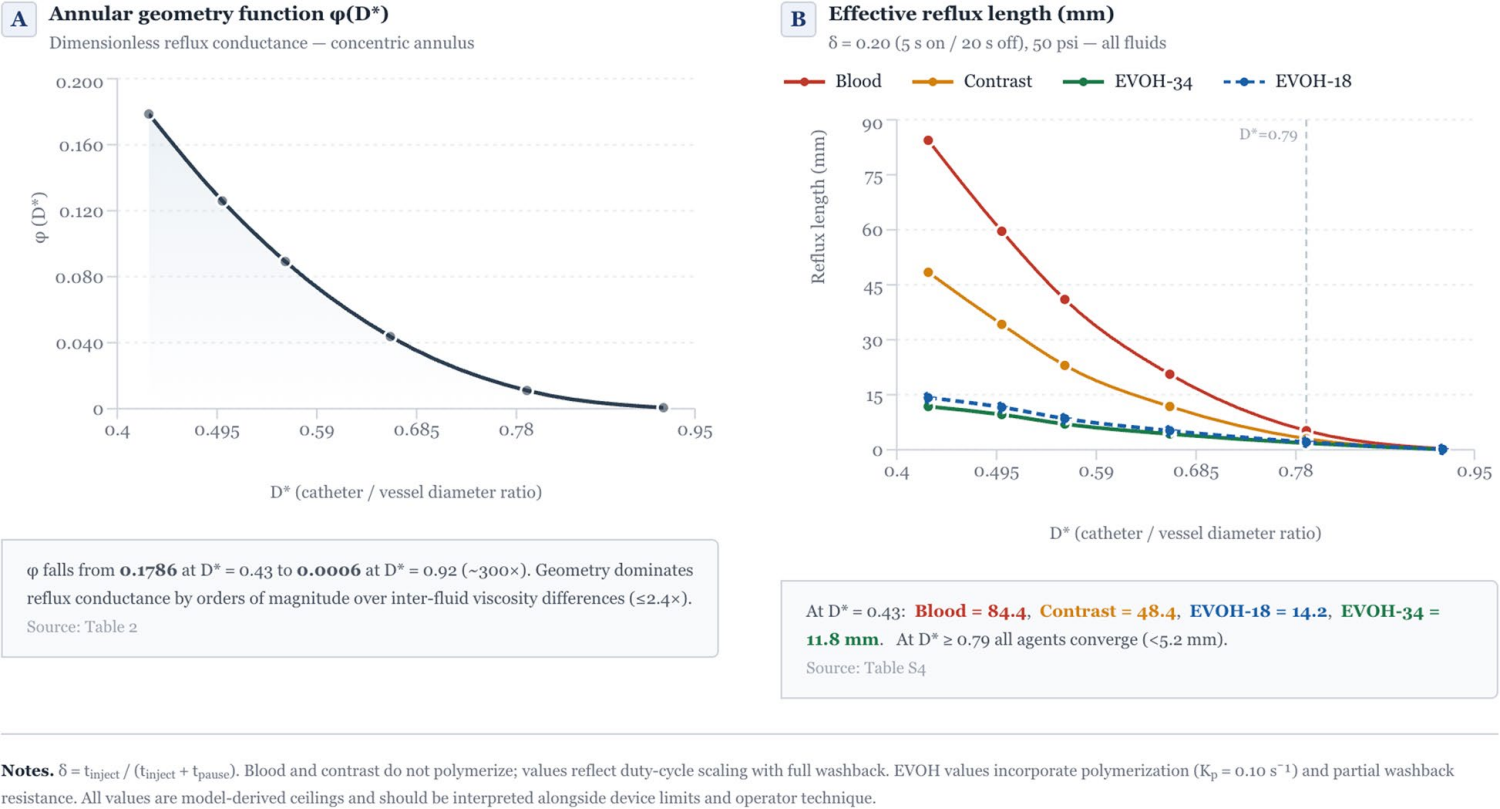
Geometry dominates reflux; intermittent injection (δ = 0.20) brings all agents into a manageable range. A: Dimensionless annular geometry function φ(D*) governing reflux conductance in a concentric annulus. φ falls from 0.1786 at D* = 0.43 to 0.0006 at D* = 0.92 (~ 300 ×), demonstrating that catheter-to-vessel fit dominates reflux by orders of magnitude. B: Effective reflux length under a realistic intermittent-injection protocol (δ = 0.20; 5 s on/20 s off) at 50 psi. At low D*, EVOH polymerization plus pauses markedly reduce reflux (e.g., EVOH-34 11.8 mm at D* = 0.43 vs contrast 48.4 mm and blood 84.4 mm). At D* ≥ 0.79, reflux is small for all agents, and inter-fluid differences are modest. *Notes: δ* = *t_inject/(t_inject* + *t_pause). Blood and contrast do not polymerize; their values reflect duty-cycle scaling with full washback. EVOH values include polymerization and partial washback resistance. All values are model-derived ceilings for the specified conditions and should be interpreted alongside device limits and operator technique*

Under a realistic duty cycle (δ = 0.20; Fig. 1B), effective reflux lengths converge to manageable values when D* ≥ 0.79, whereas at low D* the combination of polymerization and pauses reduces EVOH-34 reflux to 11.8 mm at D* = 0.43 — a 4.1-fold improvement over contrast and > sevenfold over blood (Table S4).

## Discussion

Three principal findings emerge from this analysis. First, catheter-to-vessel geometry dominates reflux: φ(D*) spans three orders of magnitude while inter-agent viscosity contributes at most 2.4-fold. This is robust to rheological uncertainty (± 25% Carreau parameter variation alters reflux ≤ 18%). When D* exceeds 0.79, reflux is under 11 mm for all agents and inter-fluid differences are clinically negligible. Second, effective EVOH viscosity under injection conditions is lower than nominal values; EVOH-18 behaves as contrast at typical shear rates. Third, EVOH polymerization provides a geometrically selective, self-regulating secondary mechanism helping most at low D* where it is needed most, while being irrelevant near wedge where geometry alone suffices.

These results provide a quantitative rationale for the push-and-wait technique: pauses reduce time-averaged driving pressure and allow precipitation to stiffen the reflux column. A duty cycle of δ ≈ 0.13–0.25 (e.g., 5 s of injection followed by 20–30 s of pause) reduced effective reflux by ~ 75–95% in our model, with diminishing returns beyond ~ 30-s pauses. In daily practice, this corresponds to injecting in short bursts and observing under fluoroscopy for reflux stabilization before resuming. Reporting protocols using δ would allow standardized comparison across centers [3].

Based on this model, catheter selection, matching the largest safely navigable microcatheter to vessel caliber, is the single most effective intervention for reflux control [2–4]. At D* ≥ 0.79 (e.g., a 2.4 F catheter in a 1 mm vessel), reflux is manageable regardless of agent. EVOH-34 combined with intermittent injection offers the greatest advantage precisely when D* is low and catheter upsizing is not feasible: at D* = 0.43 (e.g., 1.3 F in 1 mm), EVOH-34 reduced reflux 4.1-fold versus contrast. Agent choice matters most when geometry is least favorable.

However, increasing D* is not without clinical trade-offs. Advancing larger microcatheters in small-caliber or tortuous vessels increases risk of vasospasm, endothelial injury, dissection, and vessel perforation. Near-wedge positioning (D* ≥ 0.9) is not always achievable or safe, particularly in fragile intracranial vasculature. Catheter sizing should therefore be understood as a balance between reflux control and vascular safety. When catheter size is constrained by anatomy, intermittent injection with EVOH-34 provides the greatest compensatory benefit, the scenario where agent choice and injection technique become most important.

This concentric-annulus model requires further validation. Blood was modeled as Newtonian at its high-shear plateau viscosity (3.5 mPa·s); incorporating non-Newtonian rheology via Carreau or Casson models would slightly increase effective viscosity at lower shear rates, marginally reducing predicted reflux, making our assumption conservative. Real-world catheter eccentricity generally increases annular conductance, likely underestimating reflux near wedge. Absolute values reflect a 1 mm vessel example; φ depends only on D*, but reflux lengths scale with caliber. Computational fluid dynamics simulations incorporating realistic geometry and pulsatile flow, benchtop phantom validation, and retrospective clinical correlation of catheter size, vessel diameter, and reflux events represent logical next steps.

In summary, catheter-to-vessel fit is the primary, actionable determinant of reflux during liquid embolic injection, with nominal viscosity playing a secondary role. EVOH polymerization adds geometry-dependent protection maximized by intermittent injection. These model-based insights can help prioritize catheter selection and standardize technique via δ, while recognizing that coefficients and pressure ceilings require experimental validation.

## Supplementary Information

Supplementary Material 1. Figure S1. Geometric Framework.Figure S1b. Compliance Sensitivity AnalysisFigure S1b. Compliance Sensitivity Analysis. Figure S1b. Compliance Sensitivity Analysis. Figure S2. Annular Poiseuille Flow. Figure S3. Rheological Model. Figure S4. Iterative Viscosity Solution for EVOH. Figure S5. Dimensionless Parameters. Figure S6. Safety Threshold and Derived Quantities. Figure S7. Coefficient Derivation. Figure S8. Time-Dependent Viscosity Augmentation From EVOH Polymerization. Figure S9. Transient Injection Model and Duty Cycle Analysis. Table S1. Effect of Vessel Compliance on Annular Geometry Function at 50 psi. Table S2. Equilibrium Reflux Length (mm) at 50 psi, Medium Compliance — EVOH-34. Table S3. Effective Reflux Length (mm) Under Intermittent Injection Protocols (50 psi, EVOH-34, K_p = 0.10 s⁻^1^). Table S4. Effective Reflux Length (mm) at δ = 0.20, 50 psi — All Fluids.

## Data Availability

All data generated or analysed during this study are included in this published article and its supplementary information files. The analytical model is fully described in the Methods and Supplemental Material, enabling complete reproduction of all reported results.

## Abbreviations

AVM: Arteriovenous Malformation
CFD: Computational Fluid Dynamics
Ct: Craya–Curtet Number
D*: Catheter-to-vessel Diameter Ratio
dAVF: Dural Arteriovenous Fistula
DMSO: Dimethyl Sulfoxide
ECA: External Carotid Artery
EVOH: Ethylene-vinyl Alcohol Copolymer
F: French (catheter size; 1 F = 0.33 mm)
φ(D*): Annular Geometry Function
Re: Reynolds Number
δ: Injection Duty Cycle
