# Supplementary material for: Why catheter size matters more than viscosity in ethylene–vinyl alcohol copolymer reflux: an analytical study
Source: CVIR Endovasc. 2026 May 20;9:57. doi: 10.1186/s42155-026-00693-9 (PMC13190909; doi:10.1186/s42155-026-00693-9)
Supplement: Supplementary file 1 — Supplementary Material 1. Figure S1. Geometric Framework.Figure S1b. Compliance Sensitivity AnalysisFigure S1b. Compliance Sensitivity Analysis. Figure S1b. Compliance Sensitivity Analysis. Figure S2. Annular Poiseuille Flow. Figure S3. Rheological Model. Figure S4. Iterative Viscosity Solution for EVOH. Figure S5. Dimensionless Parameters. Figure S6. Safety Threshold and Derived Quantities. Figure S7. Coefficient Derivation. Figure S8. Time-Dependent Viscosity Augmentation From EVOH Polymerization. Figure S9. Transient Injection Model and Duty Cycle Analysis. Table S1. Effect of Vessel Compliance on Annular Geometry Function at 50 psi. Table S2. Equilibrium Reflux Length (mm) at 50 psi, Medium Compliance — EVOH-34. Table S3. Effective Reflux Length (mm) Under Intermittent Injection Protocols (50 psi, EVOH-34, K_p = 0.10 s⁻1). Table S4. Effective Reflux Length (mm) at δ = 0.20, 50 psi — All Fluids. [file 42155_2026_693_MOESM1_ESM.docx]

**Supplementary Materials:**

**S1. Geometric Framework.** The model considers a rigid cylindrical vessel of diameter D_h = 1 mm containing a coaxial microcatheter with outer diameter equal to its French size × 0.33 mm. The catheter-to-vessel diameter ratio is defined as D* = D_c/D_h. Catheter sizes of 1.3 F to 2.8 F correspond to D* = 0.43 to 0.92. The catheter outer diameter (OD) was used in the annular model because it sets the catheter–wall gap; inner diameter was not required for the reflux conductance calculation. The wedge boundary condition (D* = 1.0, Q* = 0) is enforced.

**S1b. Compliance Sensitivity Analysis.** To quantify the effect of vessel distensibility, we applied a linear pressure-diameter relationship: D_h(P) = D_h0 × [1 + C_v × ΔP], where D_h0 = 1.0 mm and C_v is the compliance coefficient. Published values for small muscular arteries (ECA branches, 0.5–1.5 mm caliber) range from approximately 0.5 to 2.0 %/100 mmHg. Three scenarios were evaluated: Low (C_v = 0.05%/psi); Medium (C_v = 0.15%/psi, typical ECA branch); High (C_v = 0.30%/psi, compliant or pathological vessel). For each scenario, D*_eff = D_c/D_h(P) was recomputed at 50 psi and φ(D*_eff) recalculated. Results are in Tables S1 and S2.

**S2. Annular Poiseuille Flow.** Reflux is modeled as laminar flow through the annular space between catheter and vessel wall. The volumetric flow rate is: Q_ann = (πΔP)/(8μ_eff L) × [R_o⁴ − R_i⁴ − (R_o² − R_i²)²/ln(R_o/R_i)], where R_o and R_i are vessel and catheter radii, ΔP is the pressure driving reflux, μ_eff is the effective fluid viscosity, and L is the reflux length. The dimensionless annular geometry function is: φ(D*) = 1 − (D*)⁴ − (1 − (D*)²)²/ln(1/D*).

**S3. Rheological Model.** Blood and contrast are Newtonian (viscosities 3.5 and 6.1 mPa·s at 37°C). EVOH formulations follow the Carreau model: μ_eff(γ̇) = μ∞ + (μ₀ − μ∞)[1 + (λγ̇)²]^((n−1)/2). Parameters: EVOH-18 (μ₀ = 40, μ∞ = 6 mPa·s, λ = 2.5 s, n = 0.45); EVOH-34 (μ₀ = 80, μ∞ = 8 mPa·s, λ = 3.5 s, n = 0.40). Bulk shear rate: γ̇ = 4U/D_h.

**S4. Iterative Viscosity Solution for EVOH.** For EVOH fluids, μ_eff depends on shear rate, which depends on flow velocity, which depends on μ_eff. Resolved iteratively: (1) initialize μ_eff = μ₀; (2) compute Q_ann; (3) derive velocity and shear rate; (4) update μ_eff via Carreau equation; (5) repeat until |Δμ/μ| < 1%.

**S5. Dimensionless Parameters.** The Craya–Curtet number quantifies the momentum ratio between blood and injected agent: Ct = √(ρ_b/ρ_c) × (Q_b/Q_c) × √(D_h/(D_h² − D_c²)). The Reynolds number ratio Re_b/Re_c captures relative inertial dominance of blood over the injection jet. Baseline blood flow was 1.0 mL/min; injection rate was 0.3 mL/min.

**S6. Safety Threshold and Derived Quantities.** The maximum tolerable reflux ratio was Q* = 0.05. Maximum safe injection pressure was derived by solving for ΔP at which Q* equals this threshold. Equilibrium reflux length was computed as the distance over which annular resistance balances the driving pressure at a given Q*.

**S7. Coefficient Derivation.** Empirical coefficients C and α, C₁–C₂, and C₁–C₄ require determination through CFD parametric sweeps (432 simulations: 4 fluids × 6 catheter sizes × 6 pressures × 3 baseline flows). These were not experimentally available and represent a current limitation. Post hoc fitting suggests C ≈ 0.025 and α ≈ −7.3.

**S8. Time-Dependent Viscosity Augmentation From EVOH Polymerization.** A first-order precipitation model introduces position-dependent viscosity: μ_poly(x) = μ_Carreau(γ̇) × [1 + K_p × x/v_reflux], where K_p is the precipitation rate constant (s⁻¹) and v_reflux = Q_ann/A_ann. K_p was bounded using clinical observations of EVOH solidification (30–90 seconds, approximately 5–10× viscosity increase): lower bound K_p = 0.05 s⁻¹; central estimate K_p = 0.10 s⁻¹; upper bound K_p = 0.20 s⁻¹. Integrating position-dependent viscous resistance and solving the equilibrium condition yields the quadratic: (K_p/2v_reflux) × L_poly² + L_poly − L₀ = 0, with solution: L_poly = [−1 + √(1 + 2K_p L₀/v_reflux)] / (K_p/v_reflux).

**S9. Transient Injection Model and Duty Cycle Analysis.** The injection duty cycle is δ = t_inj / (t_inj + t_pause). During injection, the reflux front advances at v_advance = Q_ann/A_ann. During pauses, blood pressure (~1.35 psi in ECA branches) drives antegrade washback at: v_retreat ≈ v_advance × (P_blood/P_inject) × (μ_EVOH/μ_blood) / (1 + K_p × t). For non-polymerizing agents, washback is fully efficient. Effective steady-state reflux length under intermittent injection was computed as: L_transient = L_poly × δ / (1 + (1−δ) × P_blood/P_inject × μ_eff/μ_blood), capturing the dual contributions of reduced time-averaged driving pressure and polymerization-augmented pause-phase resistance.

**Supplementary tables:**

**Table S1.** Effect of Vessel Compliance on Annular Geometry Function at 50 psi

| **Catheter (F)** | **Rigid φ** | **Low: D*_eff / φ / Fold** | **Medium: D*_eff / φ / Fold** | **High: D*_eff / φ / Fold** |
| --- | --- | --- | --- | --- |
| 1.3 | 0.179 | 0.42 / 0.186 / 1.04× | 0.40 / 0.198 / 1.11× | 0.37 / 0.216 / 1.21× |
| 1.5 | 0.126 | 0.49 / 0.133 / 1.06× | 0.47 / 0.146 / 1.16× | 0.43 / 0.166 / 1.32× |
| 1.7 | 0.089 | 0.55 / 0.096 / 1.08× | 0.52 / 0.109 / 1.22× | 0.49 / 0.129 / 1.45× |
| 2.0 | 0.044 | 0.64 / 0.049 / 1.13× | 0.61 / 0.059 / 1.35× | 0.57 / 0.075 / 1.72× |
| 2.4 | 0.011 | 0.77 / 0.014 / 1.28× | 0.74 / 0.020 / 1.77× | 0.70 / 0.030 / 2.70× |
| 2.8 | 0.0006 | 0.90 / 0.0014 / 2.33× | 0.86 / 0.0044 / 7.33× | 0.80 / 0.0098 / 16.3× |

*Note — Compliance effects scale with proximity to wedge. At D* ≈ 0.9, even modest distensibility can substantially increase annular conductance and reflux length; at lower D*, effects are comparatively small.*

**Table S2.** Equilibrium Reflux Length (mm) at 50 psi, Medium Compliance — EVOH-34

| **Catheter (F)** | **Rigid (mm)** | **Compliant (mm)** | **Fold** |
| --- | --- | --- | --- |
| 1.3 | 179 | 199 | 1.11× |
| 1.5 | 126 | 146 | 1.16× |
| 1.7 | 88 | 108 | 1.22× |
| 2.0 | 44 | 59 | 1.35× |
| 2.4 | 11.0 | 19.5 | 1.77× |
| 2.8 | 0.60 | 4.4 | 7.33× |

*Note — Compliance effect is most pronounced at near‑wedge (D* ≥ 0.9)* where the baseline annular gap is smallest.*

**Table S3.** Effective Reflux Length (mm) Under Intermittent Injection Protocols (50 psi, EVOH-34, K_p = 0.10 s⁻¹)

| **D*** | **δ=1.0 (cont.)** | **δ=0.50 (5s/5s)** | **δ=0.25 (5s/15s)** | **δ=0.13 (3s/20s)** | **Steady-state no polymerization** |
| --- | --- | --- | --- | --- | --- |
| 0.43 | 56.4 | 29.2 | 14.9 | 7.7 | 179 |
| 0.50 | 45.8 | 23.7 | 12.1 | 6.3 | 126 |
| 0.56 | 35.0 | 17.5 | 8.9 | 4.6 | 88 |
| 0.66 | 26.0 | 12.6 | 6.2 | 3.2 | 44 |
| 0.79 | 9.4 | 4.8 | 2.5 | 1.3 | 11.0 |
| 0.92 | 0.60 | 0.30 | 0.15 | 0.08 | 0.60 |

*Note — δ = t_inject / (t_inject + t_pause). Clinically typical protocols (δ ≈ 0.13–0.25) reduce effective reflux lengths by 75–95% compared with continuous non-polymerizing predictions.*

**Table S4.** Effective Reflux Length (mm) at δ = 0.20, 50 psi — All Fluids.

At a realistic duty cycle of δ = 0.20, reflux lengths for all agents converge to manageable values when D* ≥ 0.79, while at low D* the combined benefit of EVOH polymerization and intermittent technique reduces EVOH-34 reflux to 11.8 mm — a 4.1-fold improvement over contrast and a greater than 7-fold reduction from the continuous non-polymerizing prediction of 84.4 mm for blood. No single agent property approaches this magnitude of effect.

| **D*** | **Blood** | **Contrast** | **EVOH-18** | **EVOH-34** |
| --- | --- | --- | --- | --- |
| 0.43 | 84.4 | 48.4 | 14.2 | 11.8 |
| 0.50 | 59.6 | 34.2 | 11.6 | 9.6 |
| 0.56 | 41.0 | 23.0 | 8.5 | 7.0 |
| 0.66 | 20.6 | 11.8 | 5.2 | 4.3 |
| 0.79 | 5.2 | 3.0 | 2.1 | 1.8 |
| 0.92 | 0.28 | 0.16 | 0.13 | 0.10 |

*Note — Blood and contrast do not polymerize; values reflect duty-cycle scaling with full blood washback. EVOH values incorporate polymerization and partial washback resistance. EVOH-34 advantage over contrast at D** = 0.43 is 4.1-fold at δ = 0.20, versus 1.35-fold in the non-polymerizing steady-state model.*
